# We are operating too much

**DOI:** 10.1007/s10195-017-0471-x

**Published:** 2017-09-06

**Authors:** Nicola Maffulli

**Affiliations:** 1 Department of Musculoskeletal Disorders, University of Salerno School of Medicine, Surgery and Dentistry, Salerno, Italy; 2 Centre for Sport and Exercise Medicine, Queen Mary University of London, London, England, UK

## Abstract

With the increase of superspecialisation, there has been a recent trend for a rising number of operations for both trauma and orthopaedic ailments. This flies against the results of properly planned, well performed, adequately powered, with clinically relevant outcome measures and long enough follow-up level I studies which challenge the received wisdom that surgery is actually superior to conservative management or even supervised neglect. This editorial outlines some of these issues, and suggests that orthopaedic and trauma surgeons should actually think twice before operating on anything that comes our way.

## Editorial

Over the course of the last several years, we have witnessed major upheavals in funding and management of health care systems all over the Western world, coupled with greater stress on competence and accountability of doctors at all levels of training. Indeed, now we are considered to always be in training. In addition to employing our clinical, diagnostic and surgical skills, we are required to manage health care resources. The conundrum is that we have been given fewer and fewer such resources, that our workload is ever increasing, that, through political choices, patients have been made to raise their expectations, and expect an always perfect result.

We are surgeons, we are trained to take uncomfortable decisions, often in a less than perfect scenario, and we like operating. After all, that is why we have become surgeons! Within this daunting scenario, we are expected to always perform at our best and to be always perfect, in the face of diminishing incomes because of negative economic downturns. We nevertheless live in an imperfect world, where new techniques and implants come to light nearly every day, and an enormously large body of scientific literature is published. We have been made to love superspecialisation, and the media have embraced a culture whereby mere technical skills are regarded as being better than holistic professionalism. Ask yourself: who was more of a professional? The old professors who were able to turn their hands equally well to anything, or the young guys performing only one operation, and God forbid if a patient comes with something slightly outside their oh so very narrow area of expertise?

Accurate health care analyses predicted that the rise of superspecialisation would have resulted in an increase in the number of operations performed and corresponding health care costs, and no better patient satisfaction. We have been guilty of not following the wise advice of our elders who, as Prof Augustus Sarmiento did more than 30 years ago, advocated unity of the profession, and have instead embraced the ‘one joint, one surgeon’ dictum. Even the USA, the motherland of superspecialists, is now backpedalling, and the American Academy of Orthopaedic Surgeons is advocating to ‘Own the Bone’ as a whole.

In this climate, there has been a hard core of young and not so young clinical academics who have ploughed along, and challenged received knowledge and ancient wisdom. The underlying question they wished to clarify is whether the new and old surgical tenets were just transforming us into ‘cosmetic surgeons of the musculoskeletal system’, as Prof Sarmiento once defined modern orthopaedic surgeons, and we were just becoming prey to market forces and implants companies.

I have been lucky: I trained and developed my career in a major Western country which was a late adopter of technology, where resources have always been thin, waiting lists long, and memory longer. The old style British system selected for dedication and excellence, not just for adequate competency; it was prolonged, arduous, selective. The apprentice system did work at its best in orthopaedic surgery, and we were exposed throughout our training to many consultants who really taught us their best. In addition, cost effectiveness was always in the forefront, a healthy scepticism encouraged, and dogmas were there to be challenged. In this climate, it is not surprising that the UK has produced, over the course of the last 15 years, many appropriately powered pragmatic randomised clinical trials examining clinically relevant questions with long enough follow-up, testing the general question of whether traditional conservative management was really inferior to the wonders of modern surgery. North America has also produced some such trials, but the system over there makes randomised studies hard to conduct. In Europe and Oceania, several registries for common orthopaedic procedures have been set up, and their results are being published.

It is impossible to examine all the relevant trials and registry studies, but let me talk just about some relevant ones. If you wish, though, please challenge yourself, get on Pubmed, and search for the level I trials proving that, for example:

1. Reconstruction of the anterior cruciate ligament in active individuals is not a must (from Sweden);
2. Autologous chondrocyte transplantation for chondral defects of the knee at 2, 7 and 15 years is not superior to simple microfractures (from Norway);
3. In proximal hip fractures, modern intramedullary implants are not superior, but are more expensive and can produce more complications than good old sliding hip screws (from China);
4. Excision or trimming of meniscal tears in osteoarthritic knees in middle aged patients actually accelerates the degenerative process and does not produce any beneficial effects on pain (from Finland);
5. The use of computer assisted navigation or patient specific cutting in joint replacement decreases the number of outliers of arbitrarily set angular measurements, but does not have any impact on the longevity of the implants, while increasing the duration of the operation and its costs;
6. Aspirin is as good as, if not better than, a variety of other expensive drugs in minimising venous thromboembolic events (mainly UK and USA);
7. Minimally invasive hip arthroplasty is at best as good as traditional surgery, and in most hands clearly worse (both level I studies and registry reports from all over the world)
  …. and many more
  Details of a few such trials are apt.

The ProFHER (PROximal Fracture of the Humerus: Evaluation by Randomisation) trial is a pragmatic multicentre randomised controlled trial of surgical versus non-surgical treatment for proximal fracture of the humerus in adults [1]. The trial evaluated the effectiveness and cost-effectiveness of surgical versus standard non-surgical treatment for adults with an acute closed displaced fracture of the proximal humerus with involvement of the surgical neck. The trialists recruited 250 patients, and reported their 2-year results in a very reputable non orthopaedic journal, the Journal of the American Medical Association (JAMA) [1]. Patients with displaced proximal humeral fractures involving the surgical neck showed no significant difference between surgical and nonsurgical treatment in patient-reported clinical outcomes over 2 years after the fracture. The authors commented that these compelling results do not support the trend of increased surgery for patients with displaced fractures of the proximal humerus [2]. Many shoulder surgeons were duly horrified, and claimed that the surgeon colleagues who performed the operations in that trial were just not good enough. Well, that is patently untrue: reading the relevant protocol shows clearly that they were accomplished surgeons. Only, they were not able to do better than nature. And if there were any doubts about the 2 year results, the 5 year outcomes were just as in favour of non operative treatment (a simple sling) [3].

In 2014, again in a non orthopaedic journal, this time the British Medical Journal, the results of the DRAFFT trial were published. The trial is a multicentre two arm parallel group assessor blind randomised controlled trial with 1:1 treatment allocation. It included 461 adults with a dorsally displaced fracture of the distal radius within 3 cm of the radiocarpal joint, all requiring surgical fixation. Patients were excluded if the surgeon thought that the articular surface of the wrist joint was so badly displaced it required open reduction. Either humble Kirschner wire fixation was implemented, or the more modern fashionable expensive locking volar plates were used. Contrary to the existing literature, and against the rapidly increasing use of locking plate fixation, the trial found no difference in functional outcome in patients with dorsally displaced fractures of the distal radius treated with Kirschner wires or volar locking plates. Kirschner wire fixation, however, was cheaper and quicker to perform [4]. The DRAFFT trial has had a profound impact on English orthopaedic surgery, and has resulted in a marked change of practice. In the 5 years preceding the publication of the trial, 75% of patients with the index fracture were treated with plate fixation, and 12% with Kirschner wires. After its publication, the proportion of patients having Kirschner wires fixation rose to 42%, with a concurrent fall in the proportion having plate fixation to 48% [5].

Double versus single row fixation for repair of the rotator cuff is much debated, with very senior shoulder surgeons very sanguine for one versus the other option. The answer, however, seems to be already there. Indeed, minimal differences have been measured on clinical and functional rating scales, but these statistically significant differences seem not to be clinically relevant. Hence, the technically simpler, cheaper option should be preferred [6, 7].

Probably, no trial has shown how corporative we orthopaedic surgeons can be as Moseley et al.’s trial. In 2002, the New England Journal of Medicine published a randomised, placebo-controlled trial to evaluate the efficacy of arthroscopy for osteoarthritis of the knee in 180 patients [8]. Over a 24 month follow-up period, at no point did either of the intervention groups report less pain or better function than the placebo group. This trial was a landmark, and was widely criticised by a variety of professional bodies, who vocally questioned the ethical outlook of the surgeons who were part of the trial and the sanity of the patients who accepted to enter the trial. These results from the USA were reproduced in Canada [9] a few years later. The by then tragically defunct Alexandra Kirkley ascertained that arthroscopic surgery for osteoarthritis of the knee provides no additional benefit to optimized physical and medical therapy. Perhaps many of the orthopaedic patients with knee osteoarthritis are mad, even across the USA–Canada border!

The use of biologics in orthopaedic, and especially orthopaedic sports medicine, has become a scientifically unsubstantiated mainstay of modern practice. Unfortunately, they do not seem to be better than placebo, at least in the Achilles tendon [10] and in shoulder surgery [11]. It may be another matter in tennis elbow [12] or in knee osteoarthritis [13], but please make sure you read the accompanying learned correspondence that these other seemingly favourable trials have generated.

We are in love with technology, and with novel implants. Indeed, we are probably programmed to like new things, in order to advance and evolve. This is so very true in the arthroplasty world. In hip arthroplasty, the introduction of modular prostheses was supposed to make our life, and our patients’ life, simpler and more liveable. A shame, therefore, that these prostheses exhibit poorer survivorship than more traditional ‘outdated’ designs [14]. In this field, the metal on metal arthroplasty saga is alive and well, and should teach us something [15].

These investigations have many common features. Most of these momentous trials have not been published in orthopaedic journals. This is not an excuse for us to be ignorant of them, and not to embrace their conclusions. Also, practically all of them have been widely criticised by the relevant subspecialty societies as not being representative of real life, of not having the right outcome measures, of the fact that the patients enrolled in them were not representative of the general patient populace, or that the surgeons were not properly trained. All these criticisms sound futile, the last resort of organisations who do not wish to surrender to simple scientifically proven hard facts: in the end, instead of dominating our field of knowledge, they will empower shrewd administrators to just impose draconian cuts.

Always operating is not always right, and embracing the latest innovations should not be the standard of care. Orthopaedic surgeons are not passive recipients of industry developments, and we only need to get wrong one operation which has been shown not to be useful, to be, in the light of the present Level I evidence in favour of non-operative management in several fields, including, for example, that old friend of ours, calcaneal fractures [16], crucified forever.
